# Influence of Social Media on Perceptions and Decision-Making Regarding Cosmetic Dermatologic Procedures Among Young Adults in Saudi Arabia: A Cross-Sectional Study

**DOI:** 10.7759/cureus.111500

**Published:** 2026-06-25

**Authors:** Deema S Al Abdrabbuh, Ahmed Y Bukannan

**Affiliations:** 1 Dermatology, Royal Medical Services, Busaiteen, BHR; 2 Dermatology, Royal Medical Services, Riffa, BHR

**Keywords:** aesthetic dermatology, cosmetic dermatology, cosmetic procedures, decision-making, digital health, health communication, patient perceptions, saudi arabia, social media, young adults

## Abstract

Background: Social media has become a major source of health-related information and may significantly influence perceptions and decision-making regarding cosmetic dermatologic procedures. Young adults are among the most active users of social media and are frequently exposed to appearance-focused content; however, data examining the impact of social media on cosmetic dermatology attitudes and treatment decisions in Saudi Arabia remain limited. This study aimed to evaluate the influence of social media on perceptions and decision-making regarding cosmetic dermatologic procedures among young adults in Saudi Arabia.

Methods: A cross-sectional survey was conducted among adults aged 18-40 years residing in Saudi Arabia between January and March 2025. Data were collected using a structured, anonymous online questionnaire assessing demographics, social media use, perceptions of cosmetic dermatology, decision-making behaviors, trust in information sources, and awareness of procedural risks. A composite social media influence score was calculated to quantify the perceived impact of social media on cosmetic treatment decisions. Descriptive statistics, comparative analyses, and multivariable logistic regression were performed to identify factors associated with willingness to undergo future cosmetic dermatologic procedures.

Results: A total of 612 participants were included in the analysis. The mean age was 26.8 ± 5.4 years, and 355 participants (58.0%) were female. Frequent exposure to cosmetic dermatology content was reported by 418 participants (68.3%), while 326 (53.3%) spent more than four hours daily on social media. Social media increased awareness of cosmetic procedures (mean score = 4.23 ± 0.83) but was also perceived to contribute to unrealistic beauty standards (mean score = 4.17 ± 0.89). Nearly half of the participants reported considering a cosmetic procedure because of social media exposure (n = 302, 49.3%), and 215 (35.1%) had booked or considered booking a consultation after viewing cosmetic-related content online. Trust in dermatologist-generated content was substantially higher than trust in influencer-generated content (4.31 ± 0.74 vs. 2.54 ± 1.01). Participants with a history of cosmetic procedures demonstrated significantly higher social media influence scores than those without prior procedures (38.5 ± 5.9 vs. 31.6 ± 6.8; P < 0.001). In multivariable analysis, female sex (adjusted odds ratio (aOR): 1.84; 95% confidence interval (CI): 1.29-2.62), social media use exceeding four hours daily (aOR: 1.58; 95% CI: 1.12-2.24), frequent exposure to cosmetic content (aOR: 2.31; 95% CI: 1.61-3.32), trust in dermatologist-generated content (aOR: 1.49; 95% CI: 1.15-1.93), and higher social media influence scores (aOR: 1.72 per five-point increase; 95% CI: 1.46-2.04) were independently associated with willingness to undergo future cosmetic procedures.

Conclusions: Social media plays a substantial role in shaping perceptions and decision-making regarding cosmetic dermatologic procedures among young adults in Saudi Arabia. Greater exposure to cosmetic-related content and higher perceived social media influence were independently associated with increased willingness to pursue cosmetic treatments. Although dermatologist-generated content was viewed as highly trustworthy, concerns regarding unrealistic beauty standards and inadequate communication of procedural risks were common.

## Introduction

The rapid expansion of social media has transformed the way individuals access health information, communicate with healthcare professionals, and form opinions regarding medical and cosmetic interventions [1-5]. Platforms such as Instagram, Snapchat, TikTok, and X (formerly Twitter) have become major sources of information for young adults, offering immediate access to educational content, personal experiences, advertisements, and visual representations of aesthetic outcomes [2,3,5]. Unlike traditional health information sources, social media enables continuous exposure to appearance-related content and facilitates direct interaction between users, influencers, healthcare providers, and commercial entities [1-6].

Cosmetic dermatology has emerged as one of the medical specialties most influenced by social media [1,3,6]. The increasing popularity of minimally invasive procedures, including botulinum toxin injections, dermal fillers, laser treatments, chemical peels, and skin rejuvenation procedures, has coincided with the widespread dissemination of cosmetic content across digital platforms [2-7]. Before-and-after photographs, patient testimonials, influencer endorsements, and promotional marketing campaigns have become prominent features of online cosmetic dermatology content. These materials have the potential to increase public awareness of available treatments, improve access to educational resources, and facilitate informed discussions regarding aesthetic concerns [2-8].

However, growing concerns have been raised regarding the potential impact of social media on perceptions of beauty, body image, and cosmetic treatment expectations. The highly curated nature of online content may contribute to unrealistic aesthetic standards and increased appearance-related dissatisfaction [1,4,6]. Furthermore, cosmetic information shared on social media varies substantially in quality, accuracy, and completeness. While board-certified dermatologists increasingly use social media to educate the public and communicate evidence-based information, cosmetic content is also frequently generated by influencers, celebrities, commercial organizations, and nonmedical sources [6-10]. Consequently, users may encounter information that emphasizes treatment benefits while providing limited discussion of risks, complications, costs, or realistic outcomes.

The influence of social media extends beyond awareness and education and may directly affect healthcare-related decision-making. Previous studies have suggested that online exposure to cosmetic content can influence treatment preferences, clinic selection, perceptions of procedural safety, and willingness to undergo cosmetic interventions [10-14]. Young adults may be particularly susceptible to these influences because they represent one of the most active demographic groups on social media and are frequently exposed to appearance-focused content. Understanding the relationship between social media engagement and cosmetic dermatology decision-making is therefore increasingly important for clinicians, researchers, and public health professionals [1,3,6].

Saudi Arabia has experienced substantial growth in both social media utilization and demand for cosmetic procedures over the past decade. Young adults in the country demonstrate among the highest rates of social media engagement globally, creating an environment in which digital platforms may exert considerable influence on health perceptions and aesthetic preferences. Despite this, evidence regarding the specific impact of social media on perceptions and decision-making related to cosmetic dermatology among young adults in Saudi Arabia remains limited. Existing studies have often focused on general cosmetic surgery, individual social media platforms, or specific procedures and may not comprehensively evaluate the multiple dimensions of social media influence, including exposure, trust, risk awareness, and behavioral intentions.

A clearer understanding of how social media shapes perceptions and treatment decisions may help dermatologists develop more effective patient education strategies, address misconceptions, promote digital health literacy, and improve informed decision-making regarding cosmetic procedures. Such information may also guide professional engagement on social media platforms and support the development of evidence-based public health interventions aimed at improving the quality and reliability of cosmetic dermatology information available online.

## Materials and methods

Study design and setting

This cross-sectional survey study was conducted to evaluate the influence of social media on perceptions and decision-making regarding cosmetic dermatologic procedures among young adults in Saudi Arabia. Data were collected through an anonymous online questionnaire distributed between January and March 2025. The study targeted individuals residing in Saudi Arabia during the study period and was designed in accordance with the principles of observational survey research.

Study population and eligibility criteria

Eligible participants were adults aged 18 to 40 years residing in Saudi Arabia who were able to read and understand Arabic or English and provided informed consent electronically before participation. Individuals younger than 18 years or older than 40 years and those who did not complete the questionnaire were excluded from the analysis. To minimize duplicate responses, participants were allowed to submit the survey only once.

Sample size calculation

The required sample size was calculated using the formula for estimating a population proportion in a cross-sectional study. Assuming a 50% prevalence of social media influence on cosmetic dermatology perceptions, a 95% confidence level, and a margin of error of 5%, the minimum required sample size was estimated at 385 participants. To account for incomplete responses and improve the precision of subgroup analyses, a larger sample was targeted.

Survey instrument development

A structured questionnaire was developed following a review of the literature on social media use, cosmetic dermatology, health-related decision-making, and cosmetic procedure acceptance. The questionnaire consisted of demographic items and multiple domains assessing social media exposure, perceptions of cosmetic dermatology, decision-making behaviors, trust in information sources, and awareness of procedural risks.

Responses to attitudinal items were recorded using a five-point Likert scale ranging from 1 (strongly disagree) to 5 (strongly agree). Higher scores indicated stronger agreement with the corresponding statement. The questionnaire was reviewed by a panel of dermatologists and clinical researchers to assess content validity, clarity, relevance, and comprehensiveness. Minor modifications were implemented before survey distribution. The study questionnaire is presented in the Appendix.

Pilot testing and reliability assessment

Before study initiation, the questionnaire was pilot-tested among a sample of young adults representative of the target population. Feedback was obtained regarding item clarity, wording, readability, and completion time. Responses from the pilot phase were not included in the final analysis. Internal consistency of questionnaire domains was assessed using Cronbach's alpha coefficient, with values of 0.70 or greater considered acceptable.

Data collection

The questionnaire was administered electronically using a secure online platform. Recruitment was performed through social media channels, including Instagram, Snapchat, TikTok, X (formerly Twitter), WhatsApp, and other online networks commonly used in Saudi Arabia. Participation was voluntary, and no financial incentives were provided. Before accessing the survey, participants were presented with an information page describing the study objectives, eligibility criteria, confidentiality measures, and estimated completion time. Electronic informed consent was obtained from all participants.

Outcome measures

The primary outcome was the perceived influence of social media on cosmetic dermatology-related perceptions and decision-making. Secondary outcomes included willingness to undergo future cosmetic dermatologic procedures, trust in cosmetic information obtained through social media, awareness of procedure-related risks, and previous history of cosmetic dermatologic procedures.

A composite social media influence score was developed for this study and calculated by summing responses to selected questionnaire items assessing the influence of social media exposure, cosmetic-related content, influencer recommendations, dermatologist-generated content, online reviews, and social media-driven treatment consideration. Higher scores reflected greater perceived influence of social media on cosmetic dermatology decision-making.

Statistical analysis

Data were analyzed using IBM SPSS Statistics version 26 software (IBM Corp., Armonk, NY, USA). Continuous variables were summarized as means and standard deviations or medians and interquartile ranges, as appropriate. Categorical variables were presented as frequencies and percentages. Associations between participant characteristics and willingness to undergo future cosmetic procedures were evaluated using multivariable logistic regression analysis. Variables considered clinically relevant or demonstrating statistical significance in univariable analyses were entered into the multivariable model. Results were reported as adjusted odds ratios with corresponding 95% confidence intervals. All statistical tests were two-sided, and a P-value of less than 0.05 was considered to indicate statistical significance.

## Results

Participant characteristics

A total of 612 participants completed the survey and were included in the final analysis. The mean age was 26.8 ± 5.4 years, and 355 participants (58.0%) were female. Most respondents had attained a bachelor's degree or higher (n = 451, 73.7%), while 241 (39.4%) were students and 298 (48.7%) were employed. More than half of the participants reported spending over four hours daily on social media (n = 326, 53.3%), and 418 (68.3%) reported frequent exposure to cosmetic dermatology-related content. Previous experience with a cosmetic dermatologic procedure was reported by 135 participants (22.1%), whereas 284 (46.4%) expressed willingness to undergo a cosmetic procedure in the future (Table 1).

**Table 1:** Characteristics of the study participants. Values are presented as mean ± standard deviation (SD) or number (percentage). Social media use >4 hours/day refers to average daily use exceeding four hours. Frequent exposure to cosmetic content indicates participants reporting “often” or “very often” exposure to cosmetic dermatology-related content on social media. Percentages may not sum to 100 due to rounding. SD indicates standard deviation.

Characteristic	Total (N = 612)
Age, mean ± SD, years	26.8 ± 5.4
Female sex, No. (%)	355 (58.0)
Male sex, No. (%)	257 (42.0)
Bachelor's degree or higher, No. (%)	451 (73.7)
Student, No. (%)	241 (39.4)
Employed, No. (%)	298 (48.7)
Daily social media use >4 hours, No. (%)	326 (53.3)
Encounter cosmetic content often/very often, No. (%)	418 (68.3)
Previous cosmetic dermatologic procedure, No. (%)	135 (22.1)
Willing to undergo future cosmetic procedure, No. (%)	284 (46.4)

Social media use and sources of cosmetic dermatology information

Snapchat was the most commonly used social media platform (n = 492, 80.4%), followed by Instagram (n = 451, 73.7%) and TikTok (n = 421, 68.8%). More than half of the respondents also reported using X (Twitter) (n = 314, 51.3%), whereas Facebook use was uncommon (n = 37, 6.0%). Dermatologists represented the most frequently reported primary source of cosmetic dermatology information (n = 214, 35.0%), followed by beauty influencers (n = 167, 27.3%) and cosmetic clinics (n = 118, 19.3%). Celebrities and friends or family members were less commonly identified as primary information sources (Table 2).

**Table 2:** Social media use patterns and sources of cosmetic dermatology information. Values are presented as numbers (percentages). Participants were allowed to select more than one social media platform. The primary source of cosmetic information refers to the single most commonly used source reported by participants. Percentages are calculated based on total respondents (N = 612) unless otherwise specified.

Variable		No. (%)
Social media platforms used	Snapchat	492 (80.4)
	Instagram	451 (73.7)
	TikTok	421 (68.8)
	X (Twitter)	314 (51.3)
	YouTube	281 (45.9)
	Facebook	37 (6.0)
Primary source of cosmetic information	Dermatologists	214 (35.0)
	Beauty influencers	167 (27.3)
	Cosmetic clinics	118 (19.3)
	Celebrities	64 (10.5)
	Friends/family	49 (8.0)

Perceptions of cosmetic dermatology and social media

Participants generally reported that social media increased their awareness of cosmetic dermatologic procedures, with a mean agreement score of 4.23 ± 0.83 on a five-point Likert scale. Before-and-after photographs were perceived as influential in shaping opinions regarding cosmetic procedures (mean score = 4.01 ± 0.96), and respondents largely agreed that social media contributes to unrealistic beauty standards (mean score = 4.17 ± 0.89). Cosmetic dermatology was also viewed as having a positive effect on self-confidence (mean score = 3.92 ± 0.91). In contrast, respondents were less likely to agree that social media portrays cosmetic procedures realistically (mean score = 2.71 ± 1.03). Overall, participants perceived social media as an important factor in the normalization and increasing acceptance of cosmetic dermatologic treatments (Table 3).

**Table 3:** Participant perceptions regarding cosmetic dermatology and social media influence. Values are presented as mean ± standard deviation (SD) based on a five-point Likert scale (1 = strongly disagree, 5 = strongly agree). Higher scores indicate stronger agreement with each statement. SD indicates standard deviation.

Statement	Mean ± SD
Social media increased awareness of cosmetic procedures	4.23 ± 0.83
Before-and-after images influence perception	4.01 ± 0.96
Social media creates unrealistic beauty standards	4.17 ± 0.89
Cosmetic procedures improve self-confidence	3.92 ± 0.91
Social media portrays procedures realistically	2.71 ± 1.03
Social media encourages unnecessary procedures	3.86 ± 0.99
Cosmetic procedures are socially acceptable	4.08 ± 0.87

Influence of social media on cosmetic dermatology decision-making

Nearly half of the participants reported that social media had led them to consider a cosmetic dermatologic procedure (n = 302, 49.3%). Additionally, 267 respondents (43.6%) had searched for a cosmetic clinic after viewing related content online, and 215 (35.1%) had either booked or considered booking a consultation because of social media exposure. Cosmetic dermatologists' social media accounts were followed by 378 participants (61.8%), whereas 331 (54.1%) followed beauty influencers who promoted cosmetic procedures. Recommendations from dermatologists appeared to exert a stronger influence on treatment decisions than recommendations from influencers, with 471 participants (77.0%) agreeing that dermatologist recommendations affected their decisions compared with 241 (39.4%) for influencer recommendations. Positive online reviews also influenced treatment willingness among 394 participants (64.4%) (Table 4).

**Table 4:** Influence of social media on cosmetic dermatology decision-making behaviors. Values are presented as numbers (percentages). Items assess self-reported behaviors and perceived influence of social media on cosmetic dermatology decision-making. Influencer and dermatologist recommendation items reflect agreement or strong agreement. Participants may have engaged in multiple behaviors; therefore, categories are not mutually exclusive. Percentages are based on the total sample size (N = 612).

Variable	No. (%)
Considered a cosmetic procedure because of social media	302 (49.3)
Searched for a clinic after viewing social media content	267 (43.6)
Followed a dermatologist's account for cosmetic information	378 (61.8)
Followed a beauty influencer promoting cosmetic procedures	331 (54.1)
Booked or considered booking a consultation because of social media	215 (35.1)
Influencer recommendations affect decisions (agree/strongly agree)	241 (39.4)
Dermatologist recommendations affect decisions (agree/strongly agree)	471 (77.0)
Positive online reviews increase willingness for treatment	394 (64.4)

Trust in cosmetic dermatology information and awareness of risks

Trust in information provided by board-certified dermatologists on social media was high, with a mean score of 4.31 ± 0.74, whereas trust in influencer-generated content was substantially lower (mean score = 2.54 ± 1.01). Participants reported only moderate confidence in their ability to identify misleading information (mean score = 3.22 ± 0.96). Notably, respondents generally disagreed that social media adequately discusses procedural complications (mean score = 2.43 ± 1.07) or that cosmetic clinics provide balanced information regarding benefits and risks (mean score = 2.81 ± 1.02). Verification of information through multiple sources was relatively common, with a mean agreement score of 3.67 ± 0.94 (Table 5).

**Table 5:** Awareness and trust regarding cosmetic dermatology information on social media. Values are presented as mean ± standard deviation (SD) using a five-point Likert scale (1 = strongly disagree, 5 = strongly agree). Higher scores indicate greater trust, awareness, or agreement with the stated item. SD indicates standard deviation.

Variable	Mean ± SD
Trust in board-certified dermatologists' social media content	4.31 ± 0.74
Trust in influencer-generated content	2.54 ± 1.01
Ability to identify misleading information	3.22 ± 0.96
Social media adequately discusses complications	2.43 ± 1.07
Clinics provide balanced information on risks and benefits	2.81 ± 1.02
Verify information from multiple sources	3.67 ± 0.94

Factors associated with previous cosmetic dermatologic procedures

Participants with a history of cosmetic dermatologic procedures were more likely to be female than those without prior procedures (74.8% (101/135) vs. 53.2% (254/477), P < 0.001). They were also older (mean age = 28.9 ± 4.8 vs. 26.2 ± 5.4 years, P < 0.001), more likely to spend more than four hours daily on social media (68.1% (92/135) vs. 49.1% (234/477), P < 0.001), and more frequently exposed to cosmetic dermatology content (87.4% (118/135) vs. 62.9% (300/477), P < 0.001). Furthermore, participants who had undergone cosmetic procedures demonstrated significantly higher social media influence scores than those without prior procedures (38.5 ± 5.9 vs. 31.6 ± 6.8, P < 0.001) (Table 6).

**Table 6:** Association between participant characteristics and history of cosmetic dermatologic procedures. Values are presented as mean ± standard deviation (SD) or number (percentage). Comparisons were made using an independent-samples t-test for continuous variables and the chi-square test for categorical variables. Social media influence score represents a composite score derived from questionnaire items assessing the perceived influence of social media on cosmetic dermatology decision-making. P-values are two-sided, and statistical significance was set at P < 0.05.

Variable	Cosmetic procedure (n = 135)	No cosmetic procedure (n = 477)	P-value
Female sex, No. (%)	101 (74.8)	254 (53.2)	<0.001
Age, mean ± SD	28.9 ± 4.8	26.2 ± 5.4	<0.001
Daily social media use >4 hours, No. (%)	92 (68.1)	234 (49.1)	<0.001
Frequent exposure to cosmetic content, No. (%)	118 (87.4)	300 (62.9)	<0.001
Social media influence score, mean ± SD	38.5 ± 5.9	31.6 ± 6.8	<0.001

Predictors of willingness to undergo future cosmetic procedures

In multivariable logistic regression analysis, female sex was independently associated with willingness to undergo a future cosmetic procedure (adjusted odds ratio (aOR): 1.84; 95% confidence interval (CI): 1.29-2.62; P = 0.001). Frequent exposure to cosmetic content on social media was also associated with increased willingness (aOR: 2.31; 95% CI: 1.61-3.32; P < 0.001), as was spending more than four hours per day on social media (aOR: 1.58; 95% CI: 1.12-2.24; P = 0.009). Greater trust in dermatologist-generated social media content independently predicted willingness to undergo future treatment (aOR: 1.49; 95% CI: 1.15-1.93; P = 0.003). The strongest predictor was the social media influence score, for which each five-point increase was associated with a 72% increase in the odds of willingness to undergo a future cosmetic procedure (aOR: 1.72; 95% CI: 1.46-2.04; P < 0.001). Age was not independently associated with willingness after adjustment for other variables (P = 0.061) (Table 7).

**Table 7:** Multivariable logistic regression analysis of factors associated with willingness to undergo future cosmetic dermatologic procedures. Adjusted odds ratios (ORs) with 95% confidence intervals (CIs) are presented. Multivariable logistic regression was used to identify independent predictors of willingness to undergo future cosmetic dermatologic procedures. Variables were selected based on clinical relevance and statistical significance in univariable analysis. The social media influence score is analyzed per five-point increase. All tests were two-sided, and P < 0.05 was considered statistically significant.

Variable	Adjusted OR	95% CI	P-value
Female sex	1.84	1.29-2.62	0.001
Age (per year)	1.03	1.00-1.07	0.061
Social media use >4 hours/day	1.58	1.12-2.24	0.009
Frequent cosmetic content exposure	2.31	1.61-3.32	<0.001
Trust in dermatologist content	1.49	1.15-1.93	0.003
Social media influence score (per 5-point increase)	1.72	1.46-2.04	<0.001

## Discussion

This cross-sectional study evaluated the influence of social media on perceptions and decision-making regarding cosmetic dermatologic procedures among young adults in Saudi Arabia. Several important findings emerged. First, social media was highly prevalent among participants and represented a major source of cosmetic dermatology information. Second, exposure to cosmetic-related content was associated with increased interest in cosmetic procedures and greater willingness to pursue treatment. Third, although participants generally trusted information provided by dermatologists, they expressed considerably less trust in influencer-generated content and perceived that social media often failed to provide balanced discussions of procedural risks. Finally, greater social media influence remained independently associated with willingness to undergo future cosmetic dermatologic procedures even after adjustment for demographic and behavioral factors.

The widespread exposure to cosmetic dermatology content observed in this study reflects the increasing integration of social media into health-related information seeking. Platforms such as Snapchat, Instagram, and TikTok were used by a substantial proportion of participants, consistent with the popularity of visually oriented social media among younger populations. The predominance of these platforms may be particularly relevant in cosmetic dermatology, where treatment outcomes are frequently presented through photographs, videos, and patient testimonials. Such content has the potential to shape perceptions of attractiveness, procedural effectiveness, and treatment expectations [2-8].

A notable finding was the strong influence of before-and-after images and cosmetic-related content on participants' perceptions. Respondents reported that social media increased awareness of cosmetic procedures while simultaneously contributing to unrealistic beauty standards. This apparent paradox highlights the complex role of social media in cosmetic decision-making [2,9]. Although online platforms may improve public awareness of available treatments and facilitate access to educational content, they may also amplify appearance-related pressures and promote idealized aesthetic outcomes that are not representative of typical clinical results. The coexistence of increased awareness and heightened aesthetic expectations suggests that social media functions not only as an information source but also as a powerful social and cultural influence [3,10].

The study also demonstrated a substantial relationship between social media exposure and cosmetic treatment consideration. Nearly half of the participants reported considering a cosmetic procedure because of social media exposure, and more than one-third had considered booking a consultation after viewing online content. These findings suggest that social media may influence multiple stages of the decision-making process, including awareness, interest, information seeking, and treatment consideration. Importantly, participants with a history of cosmetic procedures reported significantly higher social media influence scores than those without previous treatment experience, further supporting the association between online exposure and cosmetic behavior.

An encouraging observation was the high level of trust placed in information provided by dermatologists compared with influencers. Participants were substantially more likely to report that recommendations from dermatologists influenced their treatment decisions than recommendations from social media influencers. These findings suggest that professional credibility remains an important determinant of health-related decision-making, even within highly commercialized social media environments. The results may reflect increasing public recognition of the importance of evidence-based information and professional qualifications when evaluating cosmetic procedures.

Despite this trust in dermatologists, participants expressed concerns regarding the quality and completeness of cosmetic information available online. Many respondents believed that social media inadequately addresses procedural complications and provides insufficient discussion of risks. This finding is clinically important because informed decision-making requires a balanced understanding of both benefits and potential adverse outcomes. The predominance of promotional content and favorable treatment outcomes on social media may contribute to an overly optimistic perception of cosmetic procedures. Consequently, there is a need for greater emphasis on transparent communication of risks, realistic expectations, and treatment limitations within digital health communication strategies [5-10].

The multivariable analysis identified several independent predictors of willingness to undergo future cosmetic procedures. Female sex, prolonged social media use, frequent exposure to cosmetic content, trust in dermatologist-generated content, and higher social media influence scores were all associated with increased willingness to pursue treatment. Notably, the social media influence score emerged as the strongest predictor, suggesting that the cumulative impact of online exposure, engagement, and perceived credibility may substantially influence cosmetic decision-making. These findings underscore the importance of considering social media as a meaningful behavioral determinant in contemporary cosmetic dermatology practice.

The findings of this study have several practical implications. Dermatologists should recognize that many patients arrive at consultations with perceptions and expectations that have been shaped by social media. Incorporating discussions regarding online information sources, digital health literacy, and realistic treatment outcomes may help improve patient education and informed consent. Furthermore, the high level of trust in dermatologist-generated content presents an opportunity for dermatologists to actively participate in social media platforms by disseminating accurate, evidence-based information and addressing common misconceptions regarding cosmetic procedures. Professional engagement in digital spaces may help counterbalance misinformation and promote safer decision-making among potential patients [5-9].

This study has several strengths. It examined multiple dimensions of social media influence, including exposure, trust, perceptions, and behavioral intentions, within a large sample of young adults from different regions of Saudi Arabia. The use of a structured questionnaire and multivariable analysis enabled a comprehensive evaluation of factors associated with cosmetic dermatology decision-making. Additionally, the focus on young adults is particularly relevant given the high prevalence of social media use within this demographic group.

Several limitations should be considered when interpreting the findings. First, the cross-sectional design precludes conclusions regarding causality, and observed associations should not be interpreted as evidence of a direct causal effect of social media exposure on cosmetic treatment decisions. Second, the study relied on self-reported data, which may be subject to recall bias and social desirability bias. Third, participants were recruited through online channels, potentially resulting in selection bias toward individuals who are more active on social media. Fourth, the assessment of social media influence was based on participant perceptions and may not fully capture the complexity of real-world online engagement. Finally, because the study was conducted in Saudi Arabia, the generalizability of the findings to populations with different cultural, social, or healthcare contexts may be limited.

## Conclusions

In this survey of young adults in Saudi Arabia, social media emerged as a significant factor influencing perceptions and decision-making related to cosmetic dermatologic procedures. Greater exposure to cosmetic-related content and higher perceived social media influence were associated with increased willingness to consider future cosmetic treatments, while information provided by dermatologists was viewed as substantially more trustworthy than content generated by influencers. At the same time, concerns regarding unrealistic beauty standards and inadequate communication of procedural risks were common. These findings highlight the growing role of social media in shaping cosmetic dermatology attitudes and underscore the importance of evidence-based digital engagement, patient education, and promotion of balanced online information to support informed cosmetic treatment decisions.

## Appendices

**Table 8:** English version of the study questionnaire. Likert scale: 1 = strongly disagree; 2 = disagree; 3 = neutral; 4 = agree; and 5 = strongly agree. ^†^ Questions 20 and 21 were answered only by participants who reported previous experience with cosmetic dermatologic procedures (Question 19 = Yes).

Section	Question No.	Item	Response options
Demographics	1	Age	18–24; 25–29; 30–34; 35–40 years
	2	Gender	Male; Female; Prefer not to say
	3	Region of residence	Central; Western; Eastern; Northern; Southern
	4	Education level	High school or below; Diploma; Bachelor's degree; Postgraduate degree
	5	Monthly income	<5,000 SAR; 5,000–10,000 SAR; 10,001–20,000 SAR; >20,000 SAR; Prefer not to answer
	6	Employment status	Student; Employed; Unemployed; Other
Social media usage	7	Which social media platforms do you regularly use?	Instagram; Snapchat; TikTok; X (Twitter); YouTube; Facebook; Other (multiple responses allowed)
	8	Average daily social media use	<1 hour; 1–2 hours; 3–4 hours; 5–6 hours; >6 hours
	9	How often do you encounter cosmetic dermatology content on social media?	Never; Rarely; Sometimes; Often; Very often
	10	Which source most frequently provides cosmetic dermatology information?	Dermatologists; Beauty influencers; Celebrities; Clinics; Friends/family; Other
Perception of cosmetic dermatology	11	Social media has increased my awareness of cosmetic dermatologic procedures.	1–5 Likert scale
	12	Cosmetic procedures appear safe based on social media content.	1–5 Likert scale
	13	Social media portrays cosmetic procedures realistically.	1–5 Likert scale
	14	Cosmetic dermatology can significantly improve self-confidence.	1–5 Likert scale
	15	Before-and-after photos on social media influence my opinion of cosmetic procedures.	1–5 Likert scale
	16	Social media creates unrealistic beauty standards.	1–5 Likert scale
	17	I believe cosmetic dermatology is becoming socially acceptable because of social media.	1–5 Likert scale
	18	Social media encourages people to seek cosmetic treatments unnecessarily.	1–5 Likert scale
Decision-making	19	Have you ever undergone a cosmetic dermatologic procedure?	Yes; No
	20^†^	Which procedure(s) have you undergone?	Botulinum toxin (Botox); Fillers; Laser treatments; Chemical peels; Microneedling; Platelet-rich plasma (PRP); Acne scar treatments; Other (multiple responses allowed)
	21^†^	Did social media influence your decision?	Not at all; Slightly; Moderately; Strongly; Very strongly
	22	Social media makes me more likely to consider cosmetic procedures.	1–5 Likert scale
	23	Influencer recommendations affect my treatment decisions.	1–5 Likert scale
	24	Dermatologists' social media accounts affect my treatment decisions.	1–5 Likert scale
	25	Positive online reviews increase my willingness to undergo cosmetic procedures.	1–5 Likert scale
	26	Before undergoing a cosmetic procedure, I would seek information from:	Dermatologist; Social media; Friends/family; Clinic websites; Other
	27	I would be willing to undergo a cosmetic procedure in the future.	1–5 Likert scale
Trust and information quality	28	Information shared by board-certified dermatologists on social media is trustworthy.	1–5 Likert scale
	29	Information shared by influencers is trustworthy.	1–5 Likert scale
	30	I can distinguish reliable cosmetic information from misleading information on social media.	1–5 Likert scale
	31	Social media adequately discusses the risks and complications of cosmetic procedures.	1–5 Likert scale
	32	Cosmetic clinics on social media provide balanced information regarding benefits and risks.	1–5 Likert scale
	33	I verify cosmetic information from multiple sources before making a decision.	1–5 Likert scale
Awareness of risks	34	Cosmetic dermatologic procedures can have complications.	1–5 Likert scale
	35	I am aware of the potential adverse effects of cosmetic procedures.	1–5 Likert scale
	36	Social media sufficiently discusses possible complications.	1–5 Likert scale
	37	Cosmetic procedures are generally low-risk when performed by qualified dermatologists.	1–5 Likert scale
	38	I would avoid a cosmetic procedure if I learned about significant risks.	1–5 Likert scale
Influencer impact	39	I have followed a cosmetic dermatologist because of social media.	1–5 Likert scale
	40	I have followed a beauty influencer who promotes cosmetic procedures.	1–5 Likert scale
	41	I have searched for a clinic after seeing social media content.	1–5 Likert scale
	42	I have booked or considered booking a consultation because of social media exposure.	1–5 Likert scale
	43	Seeing before-and-after photos increases my interest in cosmetic procedures.	1–5 Likert scale
	44	Celebrity cosmetic outcomes influence my perception of cosmetic treatments.	1–5 Likert scale
